# Occupational stress is associated with job performance among pregnant women in Japan: comparison with similar age group of women

**DOI:** 10.1186/s12884-022-05082-3

**Published:** 2022-10-05

**Authors:** Yasuka Nakamura, Yoko Sumikawa Tsuno, Aya Wada, Keiko Nagasaka, Maiko Kawajiri, Yoko Takeishi, Mikako Yoshida, Toyoko Yoshizawa

**Affiliations:** 1 Tohoku University Graduate school of Medicine, 2-1 Seiryo-machi, 980-8575 Aobaku, Sendai, Miyagi Japan; 2 Department of Health Sciences, Saitama Prefectural University, 820 Sannomiya, 343-8540 Koshigaya, Saitama Japan; 3 Bunri University of Hospitality, 311-1 Kashiwabarashindenn, 350-1336 Sayama, Saotama, Japan

**Keywords:** Absenteeism, Occupational health, Pregnant women, Job performance, Cross-sectional studies

## Abstract

**Background:**

Pregnancy results in physical and psychological changes in women; however, pregnant women hesitate to take a break from work even when they feel the need. Since working while physically ill leads to decreased job performance, it is important to determine the factors that lead to this phenomenon.

**Aim:**

To study the occupational stress associated with job performance and absenteeism of pregnant women compared with non-pregnant women.

**Methods:**

In 2019, non-pregnant and pregnant employed women in their 20–40 s in Japan completed an online survey examining job performance (Work Limitation Questionnaire - Short Form), absenteeism, occupational stress (Brief Job Stress Questionnaire), and working situations.

**Results:**

Of 918 respondents who met the inclusion criteria, 904 were included in the final analysis (454 non-pregnant and 450 pregnant women). Logistic regression analyses showed that absenteeism was significantly higher for pregnant women. However, for women who were absent, there was no significant difference between non-pregnant and pregnant women. After adjusting for attributes and working conditions, pregnant women had significantly higher (p < .001) work productivity losses than non-pregnant women, but only in the physical tasks domain; their physical stress response was also higher compared to non-pregnant women (p = .048). However, pregnant women reported significantly less interpersonal conflict stress (p < .001) and psychological stress (p = .026), as well as better workplace support as a buffering factor for stress (p = .021), than non-pregnant women.

**Conclusion:**

Clarifying the physical burden associated with pregnancy and assisting women in coordinating their work duties while considering the physical demands of pregnancy may minimize work productivity losses among pregnant women.

## Background

In recent years, health-related productivity indicators in the workplace have included absenteeism (sick leave) and job performance loss (a state in which work performance and productivity decline due to attending work with an illness or symptom). The combination of these indicators is regarded as loss of work productivity, with particular focus on the magnitude of the loss due to an illness or symptom [1]. Factors affecting job performance loss include health problems caused by occupational stress such as physical stressors, work stressors, and physical and psychological stress responses [2–4]. In previous studies addressing health-related productivity indicators, the participants were adult office workers, occupational therapists, and industrial and construction workers, and men and women were not distinguished. Pregnant women were not included in these studies.

The global labour force participation rate has declined in recent years. In Japan, women’s active participation in the labour force was recommended under the Women’s Advancement Promotion Law enacted in 2016 [5]. There are 2.31 million women in Japan who want to work, but the most common reason for not seeking employment is childbirth and childcare, accounting for 31.1% of responses [6]. However, at present, 46.9% of women in Japan leave the workforce before or after the birth of their first child [7]. Therefore, it is necessary to provide a work environment that allows women to continue working after pregnancy and childbirth.

Pregnant women are protected by laws to safeguard maternal health [8]. However, if a particular workplace does not have an atmosphere that allows both pregnancy and work, a pregnant woman may be advised to leave the workforce at the time of pregnancy [9]. As reported, the actual male managers’ comments were they felt difficulty to generally provide accurate advice on female-specific symptoms owing to limited knowledge and understanding [10], employers or bosses might not understand the actual working conditions of pregnant women and their productivity depending on the time of pregnancy. Psychological and physical stress, such as maternity harassment and the physical strain inherent in pregnancy, can lead to reduced job performance or sick leave due to work overload.

In a study on pregnant women’s absenteeism and job performance loss, 31.0-75.3% of women had taken sick leave during gestation in Norway and Denmark [11, 12]. However, there are few studies on pregnant women’s job performance decline.Japan has the lowest rate of taking paid leave in the world, and more than 60% of Japanese people believe that they feel guilty about taking paid leave [13]. In a recent study, compared to non-pregnant employed women of the same age group, absenteeism of employed Japanese pregnant women did not differ significantly due to health problems in a four-week period [14]. In other words, despite the psychological and physical burden of pregnancy, they may not take a break from work and may be working despite their pregnancy’s impact on their ability to perform work. Overwhelming psychological and physical strain due to pregnancy can lead to lower job performance, absenteeism, and even resignation as well as pregnancy complications that threaten the health of the mother and fetus.

In a Japanese working environment where pregnant women hesitate to take a break from work, if it can be clarified how pregnant women work differently from women of the same generation who are not pregnant, maternity harassment in the workplace will decrease and it will be possible to balance pregnancy and work, and ensure continued employment.

However, the specific occupational influential factors have not been reported. Therefore, in this study, the following two objectives were set:

1. To determine if pregnant women differ from non-pregnant women of the same age when it comes to occupational stress, absenteeism, and job performance.
2. To assess whether the relationships between absenteeism, job performance, and occupational stress differ between pregnant and non-pregnant women of the same age.

The findings of this study are expected to contribute to supportive practices that encourage women to manage their pregnancies and continue working without any burden being placed on their work. It is also expected to provide employers with strategies to minimize loss of work productivity when woman becomes pregnant.

## Methods

This study formed part of cross-sectional survey on the Health and Productivity of Working Women, in Japan, which targeted 1000 working women of reproductive age [14]. In this study, we use the data about occupational stress and related variables. Surveys were conducted online from April through May 2019.

Data were collected by an internet research company, Cross Marketing Group Inc., which had been granted the Privacy Mark [15].The company has an active panel of over 2.95 million people in Japan, categorized by demographics and lifestyles. The panel included subjects who were eligible for this study, screening emails were sent out to those eligible, and only those who consented to the study answered the questions.

The inclusion criteria were being a woman aged 20–44 years without any children or who were nulliparous. Women on parental leave were excluded. In Japan, the time spent by women on housework and childcare is very long compared to that of men [16], therefore women with children were excluded because of the impact having children has on occupational stress, job performance, and absenteeism.

Job performance was measured using the Work Limitations Questionnaire – Short Form (WLQ) [17], a tool that can measure the rate of decline in work performance due to physical problems in the preceding two weeks. It comprises eight items across four domains (i.e., time management, physical tasks, mental-interpersonal tasks, and output tasks). From the scores of these four domains, the productivity loss ratio is calculated as a value from 0 to 100. A percentage of 0–25 indicates at-work productivity loss in each domain. If the job type does not apply to the question items, the answer will not be applicable, and each domain and overall productivity loss ratio will not be calculated and will be treated as not being answered. Cronbach’s alphas ranged from 0.90 to 0.96 for the original [17], 0.88–0.97 for the Japanese version [18],and 0.76–0.95 in this study.

Absenteeism was calculated as the number of days the respondent was absent due to physical or mental health problems or pregnancy symptoms in the four weeks preceding the survey, calculated by half-days (0.5 days).

The Brief Job Stress Questionnaire (BJSQ) [19] was used to measure occupational stress in the four weeks preceding the survey. This scale comprises 57 items on job stressor factors (job overload, job control, interpersonal conflict, and suitable jobs) and physical and psychological stress responses with workplace support as the buffering factor. Each item consists of a 4-point scale from 1 (very true) to 4 (not true at all), with scores converted so that a high score indicates a desirable state. A person is suspected of being under stress if the number of items with the two least favorable responses for each subscale exceeds a certain level (5/11 for job overload, 2/3 for job control, 2/3 for interpersonal conflict, 2/2 for suitable jobs, 6/11 for physical stress response, 13/18 for psychological stress response, and 5/6 for workplace support). The scale’s reliability and validity have been confirmed. Also it is recommended by Japan’s Ministry of Health, Labour and Welfare as a tool for companies to measure occupational stress. Cronbach’s alphas ranged from 0.79 to 0.83 for the original Japanese version [20] and 0.86–0.90 in this study.

Data on age, marital status (married including common–law marriage/single), gestational weeks (pregnant women only), education, employment status (full-time/part-time), employee size (under/over 300 employees), shift work (yes/no), work position (any employment position/none), and working hours per week were obtained. The number of employees was divided by 300 because the Women’s Advancement Promotion Law in Japan requires companies with 301 or more regularly employed workers to establish publicly available action plans promoting the active participation of women in the workplace and to proactively analyze related issues. This means that in companies with less than 300 employees, it is possible to expect that there is no consideration or action for pregnant women, and that they are working in stressful situations that affect their productivity in some way.

Descriptive statistics on job performance, absenteeism, occupational stress, and demographic data from non-pregnant and pregnant women groups, and chi-squared tests were performed for nominal variables, while t-tests or Mann-Whitney U-tests were performed for continuous variables after confirming normality with the Kolmogorov-Smirnov test to verify these differences. We calculated Pearson’s correlation coefficient to confirm the correlation between the variables of job performance, absenteeism, job stress, and demographic data.

To clarify the association between occupational stress and job performance or absenteeism of pregnant women compared to non-pregnant women, we performed a multiple logistic regression analysis, controlling for age, education, marital status, and job condition. In order to explain where the differences exist between working pregnant women and non-pregnant women, we constructed a model in which occupational stress and productivity were taken as explanatory variables and pregnancy was the explained variable (non-pregnant women = 0, pregnant women = 1). Each item of job performance and absenteeism and all occupational stress items were used as explanatory variables. In Model 1, to explain the differences of occupational stress associated with absenteeism, the explanatory variables were absenteeism and occupational stress. In Model 2, to explain the differences of occupational stress associated with job performance, the explanatory variables were job performance and occupational stress. Finally, in Model 3, all variables of absenteeism, job performance, and occupational stress were used as explanatory variables to explain the difference between pregnant and non-pregnant women about occupational stress related to absenteeism and job performance.

In this study, a logistic regression analysis with 20 independent variables and adjustment variables is performed. In that case, a minimum of 10 samples per variable is sufficient [21], and the minimum sample size for this study is 400 samples for the pregnant and non-pregnant groups.

Analyses were performed using IBM SPSS version 26.0 (IBM Corp., Armonk, NY, USA), and statistical significance was set at p < .05.

## Results

Of the 11,162 respondents on the women’s panel who responded to the screening survey, 918 met the inclusion criteria. The final sample included 904 participants after excluding those that had the same number of responses for all question items as inappropriate responses. There were respondents from all 47 prefectures in Japan.

Participants’ general characteristics are shown in Table 1. Non-pregnant women’s age was significantly higher (mean age = 34.95, SD = 6.02) than that of pregnant women (mean age = 29.71, SD = 3.88). However, pregnant women were more likely than non-pregnant women to be married (n = 414, 92.0%, n = 98, 21.6%, respectively) and to have received up to 12 years of education (n = 233, 51.8%, n = 203, 44.7%, respectively). No significant differences were found in employment status (full-time/part-time). The average number of days a respondent was absent due to physical and mental health problems and/or pregnancy symptoms in the past four weeks was 0.79 (SD = 3.07) days for non-pregnant women and 2.29 (SD = 5.51) days for pregnant women, showing a significant difference. However, 330 (72.7%) non-pregnant women and 337 (74.9%) pregnant women had not been absent. Among those with absenteeism, non-pregnant women averaged 7.17 (SD = 8.17) days (n = 124), and pregnant women averaged 5.17 (SD = 6.26) days; no significant difference was observed (U = 6503.5 p = .334). Further, non-pregnant women had a significantly higher proportion of stress factors, such as interpersonal conflict (p < .001) and suitable jobs (p = .001). There was no significant difference regarding job overload and job control.

For the WLQ, percentages of at-work productivity loss in all four domains were significantly lower in non-pregnant women than in pregnant women (p < .001). Regarding the stress response, the number of pregnant women who required a physical stress response check was significantly higher than that of non-pregnant women (p < .001), but there was no significant difference in the psychological stress response. Non-pregnant women were significantly more likely than pregnant women to check for workplace support as a buffering factor (as shown in Table 2).

**Table 1:** Demographic data of pregnant and non-pregnant women

	Non-pregnant women (n = 454)		Pregnant women (n = 450)		
	**Mean**	**SD**	**Mean**	**SD**	**p value**
	**﻿(range)**		**(range)**		
Age	34.95	6.02	29.71	3.88	< .001^a^
	(22–44)		(21–42)		
Gestational weeks			23.17	7.17	
			(10–40)		
	**n**	**%**	**n**	**%**	**p value**
Married (including common–law marriage)	98	21.6%	414	92.0%	< .001^b^
Educated up to 12 years	203	44.7%	233	51.8%	0.039 ^b^
Full-time employment	226	49.8%	238	52.9%	0.350 ^b^
Over 300 employees	135	29.7%	141	31.3%	0.614 ^b^
Shift work	93	20.5%	106	23.6%	0.297 ^b^
Having job position	43	11.3%	34	8.3%	0.186 ^b^
Work more than 40 h a week	274	60.4%	250	55.6%	0.082 ^b^

^a^Mann-Whitney U-test, ^b^ Chi-squared tests

**Table 2:** Comparison of productivity of pregnant and non-pregnant women

	Non-pregnant women (n = 454)		Pregnant women (n = 450)		
	**Mean**	**SD**	**Mean**	**SD**	**p value**
	**(range)**		**(range)**		
Absenteeism	0.79	3.07	2.29	5.51	< 0.001 ^a^
	(0–28)		(0–28)		
WLQ_Productivity loss	3.85	4.72	5.83	5.61	< 0.001 ^a^
	(0–24.9)		(0–24.7)		
WLQ_Time management	11.4	21.1	19.2	27.6	< 0.001 ^a^
	(0–100)		(0–100)		
WLQ_Physical tasks	14.6	19.1	27.2	23.0	< 0.001 ^a^
	(0–100)		(0–100)		
WLQ_Mental–interpersonal tasks	17.4	21.9	23.3	23.4	< 0.001 ^a^
	(0–100)		(0–100)		
WLQ_Output tasks	12.3	20.4	19.4	25.6	< 0.001 ^a^
	(0–100)		(0–100)		
	**n**	**%**	**n**	**%**	**p value**
BJSQ					
Job overload	175	38.5%	182	40.4%	0.586 ^b^
Job control	187	41.2%	188	41.8%	0.893 ^b^
Interpersonal conflict	153	33.7%	96	21.3%	< 0.001 ^b^
Suitable jobs	113	24.9%	71	15.8%	0.001 ^b^
Psychological stress response	103	22.7%	86	19.1%	0.192 ^b^
Physical stress response	81	17.8%	125	27.8%	< 0.001 ^b^
Workplace support	252	55.5%	165	36.7%	< 0.001 ^b^

WLQ: Work Limitations Questionnaire – Short Form; BJSQ: The Brief Job Stress Questionnaire

^a^Mann-Whitney U-test, ^b^ Chi-squared tests

Using multiple logistic regression analysis with pregnant women, we clarified the type of occupational stress that affects pregnant women’s productivity. The findings regarding occupational stress are shown in Table 3. Regarding attributes and employment factors, shift work was excluded because there was no significant difference between non-pregnant women and pregnant women. Finally, age, marital status, education years, employment status, employee size, position, and a 40-hour workweek were used as control variables. Additionally, we used only four domains of the WLQ for job performance because there was a significantly strong correlation between the total loss ratio of the WLQ and each of the four domains (r = .827–0.918, p < .01). For the four domains, no correlation exceeding 0.8 was observed. In addition, it allows for a detailed evaluation of job performance in time management, physical tasks, mental-interpersonal tasks, and output tasks, which cannot be assessed by the total score.

In Model 1, pregnant women had higher odds of absenteeism (OR = 1.077, p = .014, 95% CI [1.015–1.143]) and physical stress response (OR = 2.221, p = .004, 95% CI [1.226–4.024]). Meanwhile, for workplace support, pregnant women had lower odds (OR = 0.514, p = .004, 95% CI [0.326–0.808]).

In Model 2, when occupational stress and job performance were inputted together, pregnant women only had higher odds for physical tasks (OR = 1.040, p < .001, 95%CI [1.023–1.057]), and those for time management, mental-interpersonal tasks, and output tasks were not significant. For occupational stress, pregnant women had higher odds of physical stress response (OR = 2.050, p = .038, 95% CI [1.040–4.039]). In contrast, workplace support (OR = 0.548. p = .018, 95% CI [0.332–0.903]), interpersonal conflict (OR = 0.363, p < .001, 95% CI [0.203–0.648]), and psychological stress response (OR = 0.476, p = .029, 95% CI [0.244–0.928]) showed lower odds for pregnant than non-pregnant women.

In Model 3, occupational stress, absenteeism, and the four domains of the WLQ were analyzed together. As a result, absenteeism was no longer significant, and among the four domains of the WLQ, time management, mental-interpersonal tasks, and output tasks were not significant, and only physical tasks had a higher odds ratio for pregnant women (OR = 1.040, p < .001, 95% Cl [1.023–1.057]). For occupational stress, the same variables as in Model 2 were significant (physical stress: OR = 2.054, p = .038, 95% Cl [1.042–4.050]; interpersonal conflicts: OR = 0.361, p < .001, 95% Cl [0.202–0.646]; psychological stress response: OR = 0.478, p = .031, 95% Cl [0.245–0.934]; workplace support: OR = 0.546, p = .018, 95% Cl [0.331–0.902]).

**Table 3:** Odds ratios for productivity and job stress by pregnancy (= 1) and non-pregnancy (= 0)

	Model 1					Model 2					Model 3					
	ORs	95% CI			p value	ORs	95% CI			p value	ORs	95% CI			p value	
BJSQ																
Job overload	0.83	0.51	–	1.35	0.447	0.67	0.39	–	1.17	0.161	0.67	0.39	–	1.17	0.163	
Job control	1.36	0.84	–	2.21	0.217	1.50	0.88	–	2.56	0.136	1.50	0.88	–	2.57	0.135	
Interpersonal conflict	0.43	0.25	–	0.72	0.002	0.36	0.20	–	0.65	< 0.001	0.36	0.20	–	0.65	< 0.001	
Suitable jobs	0.71	0.40	–	1.27	0.249	0.91	0.48	–	1.72	0.762	0.91	0.48	–	1.73	0.770	
Psychological stress response	0.61	0.33	–	1.123	0.115	0.48	0.24	–	0.93	0.029	0.48	0.25	–	0.93	0.031	
Physical stress response	2.25	1.25	–	4.08	0.007	2.05	1.04	–	4.04	0.038	2.05	1.04	–	4.05	0.038	
Workplace support	0.51	0.32	–	0.80	0.004	0.55	0.33	–	0.90	0.018	0.55	0.33	–	0.90	0.018	
Absenteeism	1.08	1.02	–	1.14	0.014						1.01	0.99	–	1.01	0.864	
WLQ_Time management						1.00	0.99	–	1.01	0.996	1.00	1.02	–	1.06	0.978	
WLQ_Physical tasks						1.04	1.02	–	1.06	< 0.001	1.04	0.98	–	1.01	< 0.001	
WLQ_Mental–interpersonal tasks						1.00	0.98	–	1.01	0.554	1.00	0.98	–	1.01	0.547	
WLQ_Output tasks						0.99	0.98	–	1.01	0.432	0.99	1.08	–	1.17	0.424	

Control variables: age, marriage status (inc. common–law marriage), educated up to 12 years, full-time employment, over 300 employees, having a job position, working more than 40 h a week

ORs: Odds Ratios, WLQ: Work Limitations Questionnaire – Short Form, BJSQ: The Brief Job Stress Questionnaire

## Discussion

This study found that pregnant women had significantly more absenteeism in the four weeks preceding the survey than non-pregnant women, according to the univariate analysis. However, about 70% of the women were not more absent. In the group of women who were absent, there was no significant difference between non-pregnant and pregnant women, although non-pregnant women had *more* days of absenteeism. Thus, being ill will cause a woman to be absent from work, whether pregnant or not. In our ongoing series of studies, we have not yet investigated the specific reasons and type of leave (sick leave, medical checkups, family reasons, etc.) for absenteeism. In Japan, it is common to take paid leave during illness instead of sick leave, making it difficult to understand what kind of health problems cause absenteeism [22]. However, to secure a female labour force, it will be necessary to clarify the specific causes of absenteeism due to poor physical conditions—including pregnancy-related symptoms—so that measures can be taken to prevent absenteeism.

This study is the first to clarify the association of pregnancy-related physical burden with the job performance of women who work. Pregnant women reported higher odds for physical tasks domain and physical stress response even after adjusting for age and employment status. That is, pregnant women had higher physical stress response and productivity loss related to physical tasks than non-pregnant women. However, they received more support at the workplace and had fewer interpersonal conflicts and psychological stress responses with a lower odds ratio than non-pregnant women. Women can feel burdened by the significant physical changes that occur over the course of pregnancy. This is especially true for women expecting their first child, as it is their first experience with such substantial physical changes, and they may not know how to manage them. Physical burden may cause employment-related stress and increase productivity loss. Physical load, such as the degree of back pain in nurses [23] and health problems in workers who are already physically burdened, affects job performance loss [2]. Interestingly, pregnant women were no different from non-pregnant women in the other three domains such as time management, mental-interpersonal tasks, and output tasks. Pregnancy causes various biological/physiological [24], psychological, and social changes [25]. For example, after having children, women’s time management skills improve [26], although pregnant women may be trying to do things efficiently in more limited time. The problem, however, is that pregnant women also tend to work excessively due to guilt [27] and in order to maintain their image [28] despite the physical burden. This can cause pregnancy complications and psychological distress, leading to sickness absenteeism [29, 30].

However, support at workplaces, interpersonal conflicts, and psychological stress responses were lower in non-pregnant women. In Japan, a social system of maternity health care guidance item contact cards is used to ensure that when a pregnant woman receives guidance from a doctor, her employer can respond appropriately according to the card’s contents [8]. Pregnant women can avail themselves of these benefits. These systems are especially important because some pregnant women feel that disclosure of their pregnancy could lead to them being removed from their charge, advised to quit, or could lead to feelings of causing trouble to others [27, 31]. Therefore, by reporting their pregnancy to their workplaces and availing themselves of such social resources, pregnant women can receive workplace support and minimize psychological stress at work. However, as this study did not investigate pregnant women’s use of social resources, the interpretation of the results is limited.

The annual economic loss of a woman retiring after giving birth was estimated to be 1174.1 billion yen ($10.8 billion) [32]. The same report also indicated that regular female employees retiring due to childbirth, even if they are re-employed afterwards, have an average difference of more than 80 million yen in their lifetime annual incomes. If a woman leave the workforce during her pregnancy, it will be a loss for both the company and the woman.

Because many women leave the workforce due to difficulties in balancing pregnancy and work or maternity harassment, providing support so that they may continue working and improving support for their physical burden can prevent them from retiring. Specifically, bosses who manage working pregnant women should be obliged to review their workload and ensure that they are not burdened with work that requires standing for a long time, handling heavy objects, and tasks that require leaning forward. Additionally, modifications to the workspace are recommended, including adding stools for sitting, stopping work every 60 min to allow for a stretching break, and providing pregnant women with supportive and shock absorbent shoes depending on the type of office flooring. With these measures, it is possible to achieve the government’s target of a continuous employment rate of 70% for women before and after the birth of their first child by 2025 [33].

## Strengths and limitations

The pregnant women in this study were working and did not include those who left the workforce after pregnancy. Therefore, all participants could continue working after becoming pregnant. As reported reasons for leave the workforce due to pregnancy include physical condition problems and disadvantageous treatment due to pregnancy [31, 34], it is possible that factors other than occupational stress are also associated with workplace productivity. Furthermore, it is possible that the use of social systems also had an effect, although it was not examined in this survey. It has been reported that job performance during pregnancy is lower in early pregnancy, increases toward the middle of pregnancy, and then decreases toward the end of pregnancy [14, 35]. Therefore, The period of pregnancy should be taken into account even if the woman is pregnant.

Additionally, as this was a cross-sectional study, we could not measure and compare differences in the absenteeism and job performance of each woman before and after they became pregnant. However, the analysis was conducted by adjusting for demographic characteristics, such as age and employment status, and reasonable results were obtained to some extent. Future studies should investigate occupations that are difficult to substitute, such as medical care professionals, health and welfare workers, and teachers, given that these types of occupation are related to productivity [36].

## Conclusion

We investigated the association of occupational stress with the work productivity of pregnant and non-pregnant women of reproductive age. Absenteeism was significantly higher in pregnant women, but there were no differences within the absentee group, and there was no significant difference in the number of days a respondent was absent between those absent due to pregnancy-related symptoms and those absent due to other health problems. Additionally, in the condition of job performance loss, also referred to as presenteeism, the physical stress response was significantly higher in pregnant women than in non-pregnant women only in the domain of physical tasks, but the stress factor for interpersonal conflicts was lower in pregnant women. Further, pregnant women had lower psychological stress, and workplace support was a buffering factor. These findings indicate that pregnant women are physically burdened, and by developing concrete measures to mitigate physical burden in the workplace, pregnant women’s work productivity may improve.

## Data Availability

The data are not yet available as they are still being analyzed for additional research. Please contact the corresponding author for requests.
